# Factors associated with procedure‐related cardiac tamponade

**DOI:** 10.1002/joa3.12941

**Published:** 2023-10-11

**Authors:** Naoya Kataoka, Teruhiko Imamura

**Affiliations:** ^1^ Second Department of Internal Medicine University of Toyama Toyama Japan

To editor:

Deshpande and colleagues conducted a rigorous investigation into the incidence of cardiac tamponade following invasive cardiac procedures, utilizing a nationwide inpatient sample database.^1^ Their compelling findings demonstrated that the occurrence of cardiac tamponade during cardiac electrophysiology procedures ranged from 3.4% to 7.0%, with a corresponding in‐hospital mortality rate of 2.2% in real‐world clinical settings. However, several pertinent concerns have been raised regarding the scope of their study.

Cardiac tamponade is characterized by unstable hemodynamics arising from progressive pericardial effusion and restricted cardiac diastole. In the practical realm, clinicians frequently encounter patients presenting with mild pericardial effusion alongside stable hemodynamics, a scenario that can often be managed through medical interventions without resorting to invasive procedures. The utilization of an international database by the authors raises the possibility that their cohort may have included individuals with pericardial effusion and stable hemodynamics, that is, those who did not manifest cardiac tamponade. A critical question that emerges is whether all participants in their cohort underwent interventional pericardial drainage, a query warranting clarification.

An intriguing observation made in their study is the comparatively higher incidence of cardiac tamponade after catheter ablation, approximately 5%,^1^ as contrasted with a lower incidence, below 1%, reported in the Japanese context.^2^ It would be insightful for the authors to conduct a comparative analysis between countries, delving into the incidence of procedure‐related complications, including cardiac tamponade. This analysis could provide valuable insights into the underlying reasons for such discrepancies.

It is well‐established that the incidence of procedure‐related complications is significantly influenced by a myriad of factors, including procedural duration, operator experience, and institutional case volume.^3^ These variables may serve as confounders impacting the primary outcomes in their study. Notably, the study highlights that older patients undergoing atrial fibrillation ablation exhibited a higher mortality rate.^1^ Should any modifiable factors be identified, intervening in such variables may offer a promising avenue to mitigate the incidence of complications, thereby enhancing patient safety and clinical outcomes.

## CONFLICT OF INTEREST STATEMENT

Authors declare no conflict of interests for this article.
